# Interrupted Aortic Arch in an Adult with Polycystic Kidney Disease

**DOI:** 10.1155/2013/404710

**Published:** 2013-06-09

**Authors:** Ayşe Şeker Koçkara, Mansur Kayataş, Can Huzmeli, Ferhan Candan, Cesur Gümüş

**Affiliations:** ^1^Department of Nephrology, Cumhuriyet University Medical School, 58140 Sivas, Turkey; ^2^Department of Radiology, Cumhuriyet University Medical School, 58140 Sivas, Turkey

## Abstract

Autosomal dominant polycystic kidney disease (ADPKD) is the most common hereditary kidney disease and is responsible for 8–10% of patients with end-stage renal failure. The major extrarenal complications of ADPKD are cardiovascular abnormalities. Interrupted aortic arch (IAA) is a lethal congenital cardiac abnormality seen with a frequency of 3/1000000 births and is defined as a segment of the arcus aorta being atresic. In the literature, there are no any reports showing that polycystic kidney disease and interrupted aortic arch occur together. In this study, we present a rare case in which the patient has polycystic kidney disease and IAA together and discuss whether IAA is a complication of ADPKD.

## 1. Introduction

ADPKD is considered as a systemic disease as cysts are developed in some other organs besides the kidneys, and some other pathologies belonging to various systems could also be seen in it. The major extrarenal complications of ADPKD are cardiovascular abnormalities which increase morbidity and mortality rates. Among the cardiovascular complications, hypertension and heart valve disorders are the most common ones. Aortic root dilatation, bicuspid aortic valve, and coarctation of the aorta can also be seen [1–3]. Despite its clinical importance, little is known about the pathogenesis of the cardiovascular manifestations associated with ADPKD. The role of mutations in PKD1 and PKD2 genes in aortic dissection, dilatation, and coarctation has been shown in animal experiments. It has been suggested that the polycystines which are the products of these genes could shed light on the pathogenesis of the disease [4–8].

Interrupted aortic arch is a congenital malformation with three distinct types, characterized by complete luminal dissociation between the ascending and descending aorta, and accounting for less than 1% of all cases of congenital heart diseases [9]. Although a diagnosis of infancy is primarily considered, there have been more than 30 cases reported in the adult population [10]. The IAA found in our patient is not an isolated anomaly as it is accompanied by ADPKD. The IAA diagnosed at an adult age might have been developed as a result of the progression of aortic coarctation. Therefore, IAA might be one of the aortic complications of ADPBH although its pathogenetic mechanism could not yet be proved.

## 2. Case

**Figure 1:**
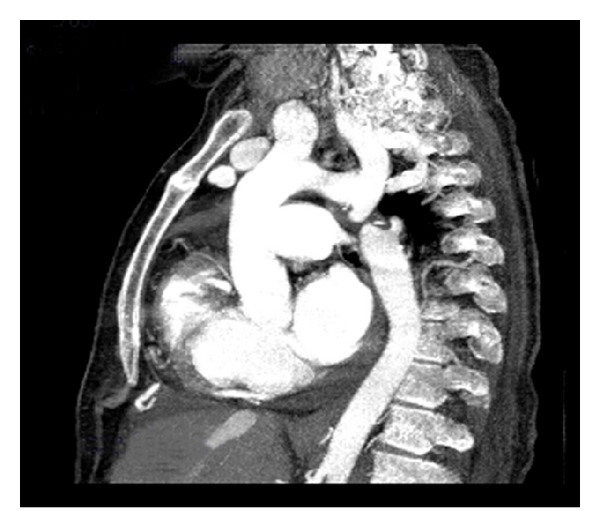
CT angiography showing type A interrupted aortic arch distal to the left subclavian artery.

**Figure 2:**
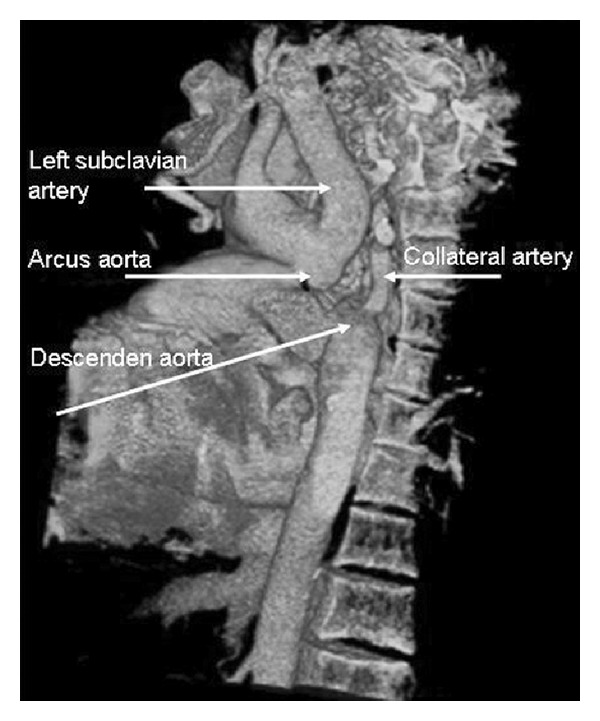
Three-dimensional reconstruction image showing type A interrupted aortic arch with collateral artery near the descending aorta.

A 69-year-old woman patient, under a routine, three times a week hemodialysis programme for 8 years, with a history of polycystic kidney disease accompanied by chronic renal failure (CRF) and chronic obstructive pulmonary disease (COPD), complained of a chest pain and dyspnea while being evaluated during one of her dialysis sessions. In the physical examination, her blood pressure (BP) in the right upper extremity was found to be 140/80 mmHg and heart rate 78/min, and heart and breath sounds were normal. Her electrocardiogram showed sinus rhythm. Cardiac enzymes observed for the purpose of excluding the diagnosis of acute coronary syndrome are found within the normal range. Echocardiographic evaluation showed left ventricular hypertrophy, dilatation in the right heart cavities, pulmonary hypertension, 2-3° tricuspid regurgitation, 1° aortic regurgitation, and minimal pericardial effusion. In BP measurements, while blood pressures in both upper extremities were found to be at the hypertension level (BP: 150/90 mmHg), the lower extremity pressures were found to be at low levels (BP: 110/70). Hence, spiral thorax CT angiography was applied to the patient, who was suspected to have aortic coarctation. It was seen in her report that the descending aorta was interrupted in a 1 cm segment starting from the subclavian arterial exit. It was observed that the distal of the descending aorta was being filled with internal mammarian and paravertebral collaterals (Figures 1 and 2). Coronary arteries were also evaluated in CT angiography, and no critic lesions were observed. The patient was diagnosed with interrupted aortic arch type A, and with the decision of a common meeting of cardiology and cardiovascular surgery, it was agreed to keep the patient under control with medical treatment (metoprolol 1 × 50 mg, ramipril 1 × 5 mg, and acetylsalicylic acid 1 × 100 mg). The patient, whose complaints have been considerably reduced, is still getting hemodialysis treatment in our center.

## 3. Discussion

 ADPKD occurs as a result of mutations in two different genes. The gene responsible for the 85% of the cases is the polycystic kidney disease 1 PKD1 gene located on the shorter arm of the 16th chromosome, and the other gene responsible for the rest of the cases is the polycystic kidney disease 2 PKD2 gene located on the longer arm of the 4th chromosome [11]. PKD1 and PKD2 genes encode the polycystin 1 and polycystin 2 proteins. Polycystin, form a calcium-permeable ion channel complex that regulates the cell cycle and the function of the renal primary cilium. Both genes are expressed in the various organs and tissues including smooth muscle cells and endothelial cells of the blood vessel [12]. 

 The association of ADPKD with berry aneurysms, vertebral artery dissection, and abdominal aortic aneurysms, in addition to thoracic aortic dissection, suggests an abnormality of the structure of the arterial wall. Connective tissue abnormalities have been observed in ADPKD. Extracellular matrix components such as collagen type IV, proteoglycan, fibronectin, undulin, and tenascin have all been demonstrated to be abnormal in the pattern of deposition and proportion [13–15].

 Studies on the mouse PKD1 gene may provide valuable insights into PKD1 functions because of the close similarity between the human and murine gene and gene product. In normal development, murine PKD1 is expressed at high levels from the morula stage and is detected in all neural crest cell derivatives, including adult brain, aortic arch, cartilage, and mesenchymal condensation [16, 17].

 Several mouse models have been established to study ADPKD using targeted disruption of the PKD1 or PKD2 genes. Homozygous deletion of PKD1 (PKD1^del34/del34^ and PKD1^null/null^) is embryonically lethal, with numerous large cysts in kidney and pancreas. PKD1^L/L^ embryos die primarily of cardiovascular defects (e.g., edema, vascular leaks, and rupture of blood vessels) with renal and pancreatic cystic development by embryonic day 15.5 (E15.5) and E13.5, respectively, [4–6].

 Mice with a PKD2 null mutation also die between E13.5 and E18 with cardiac defects and hemorrhages [7]. ADPKD-associated vascular manifestations, such as aortic root dilatation, coarctation of aorta, and abdominal aortic aneurysm, are also associated with the mutation in PKD1 [6, 8, 14]. Therefore, it seems likely that the function of the polycystins is essential to the development and maintenance of the myoelastic structural organization of the vasculature. 

 IAA presents as a severe congestive heart failure in the neonatal period, and 90% of affected neonates die at a median age of 4 days. Patent ductus arteriosus is needed for perfusion of the descending aorta. Death within the first days of life is due to closure of the patent ductus arteriosus. Survival into adulthood is dependent upon the development of substantial collateral circulation. In adult patients, the most presenting symptom is refractory hypertension to medical management. Other common presenting symptoms are claudication and limb swelling [18–20]. However, occasionally, asymptomatic cases at an adult age could also be observed; these patients, as in the cases having aortic coarctation with light symptoms, are often diagnosed either by coincidence or when a hypertension etiology is being searched [21]. 

 In our patient, IAA was found by coincidence while she was being examined because of her complaints of chest pain and dyspnea. But, probably, the complaints of our patient are nonspecific findings occurring as a result of hypertension and heart valve disorders accompanying the polycystic kidney disease. Hossack and his friends report that there are cardiovascular symptoms in all of the cases having polycystic kidney disease. It has been reported in studies on the subject that the most common symptoms in the patients with polycystic kidney disease are atypical chest pain and palpitation and the most common electrocardiogram finding is the left ventricular hypertrophy [22–24]. 

 In a review published in 2011, 37 interrupted aortic cases diagnosed at an adult age were reported [10]. Seven of them were reported in Turkey. The majority of patients in the literature underwent surgical correction. Considering the advanced age of our patient, together with her CRF and COPD history, an operation is thought to be risky, and with the decision of the council, medical therapy has been suggested.

 There is no other case reported in the literature in which polycystic kidney disease and interrupted aortic arch are observed together. Perhaps, a possible relationship might have been overlooked because the lower and upper extremities examination is ignored during the routine examination in ADPKD cases. Therefore, this relationship will be revealed more clearly if the pulse and BP measurements are taken into account, and one of the suitable monitoring methods are employed in suspected cases.

 Patients diagnosed with interruption of the aortic arch in adulthood might be displaying progression of undiagnosed coarctation of aorta. In other words, interrupted aorta could be considered as the severe form of aortic coarctation. ADPBH is regarded as one of the genetic diseases affecting the aorta [25]. In addition to aortic dilatation, dissection, and coarctation, interruption could also be one of the cardiovascular complications of ADPBH.
